# The correlation between modifications to corneal topography and changes in retinal vascular density and retinal thickness in myopic children after undergoing orthokeratology

**DOI:** 10.3389/fmed.2023.1166429

**Published:** 2023-06-29

**Authors:** Yan Lian, Weiwei Lu, Aiqin Xu, Renai Chen, Qingqing Lu, Weihe Zhou, Lili Mei, Wanqing Jin

**Affiliations:** ^1^National Clinical Research Center for Ocular Diseases, Eye Hospital, Wenzhou Medical University, Wenzhou, China; ^2^School of Ophthalmology, Optometry, Wenzhou Medical University, Wenzhou, Zhejiang Province, China; ^3^The First People’s Hospital of Aksu District in Xinjiang, Aksu City, China

**Keywords:** orthokeratology, OCTA, retinal thickness, relative corneal refractive power shift, retinal vascular density

## Abstract

**Purpose:**

This study aimed to investigate the relationship among changes in corneal topography, retinal vascular density, and retinal thickness in myopic children who underwent orthokeratology for 3 months.

**Method:**

Thirty children with myopia wore orthokeratology lenses for 3 months. Using optical coherence tomography angiography (OCTA), the retina was imaged as 6 × 6 mm en-face images at baseline and 3 months after orthokeratology. Cornea data was acquired by topography and analyzed by customer MATLAB software. The cornea was divided into 3 zones and 9 sectors. The relative corneal refractive power shift (RCRPS) was used in this study. Changes in retinal vascular density (RVDC) and retinal thickness change (RTC) were associated with RCRPS by using spearman test. Statistical significance was set at *p* < 0.05.

**Result:**

A significant correlation was observed between the RVDC and the RCRPS in many regions (the r was 0.375 ~ 0.548, all *p* value <0.05). Significant positive correlations were found between RVDC in inner and outer temple regions with RCRPS at inner and outer nasal sectors. There were no significant correlations between RTC and RCRPS in other sectors except in the central cornea and the outer nasal retina (*r*:0.501, *p*:0.006). At baseline and 3 months after wearing the orthokeratology lens, no significant differences in the retinal microvasculature or thickness (*p* > 0.05) were observed at any regions.

**Conclusion:**

The correlation between the cornea and the retina was observed after orthokeratology. Cornea changes may affect regional retinal responses accordingly，which may explain how orthokeratology delays myopia progression partially.

## Introduction

The prevalence of myopia has increased rapidly, particularly in Asia (1). Studies have shown that high myopia incidence is associated with an increased prevalence of myopic maculopathy (2). Increasing one myopic diopter will increase myopic maculopathy risk by 67% (3). Reduced myopic maculopathy is one of the benefits of myopia control (4). Orthokeratology has been proven to be a safe and effective method for myopia control (5, 6). Orthokeratology involves changing the cornea morphology using reverse geometry designs, causing the central and mid-peripheral corneas to be flat and steep, respectively (7, 8). Orthokeratology delays myopia progression by changing the corneal refractive power, causing peripheral defocus in the eye (8–12).

In addition, orthokeratology affects the retina and choroid (13–15), which were found to be thickened through optical coherence tomography (OCT) after wearing orthokeratology lenses (13, 16). According to Chen’s study, after wearing orthokeratology lenses for 6 months, the retinal vasculature did not change significantly (17). Despite the differences in their results, some studies proved that OCT angiography (OCTA) is a valuable clinical tool for evaluating retinal vascular morphologies, especially the retinal veins (18–20). Altering retina ganglionic cell signaling when retina defocus occurred was thought to be the possible mechanisms for myopia regulation (21).

In subjects wearing OK lenses, corneal changes after wearing lens were the optical factor of retinal defocus. A more specific and direct way is the relative corneal refractive power (RCRP), which was described by Wang et al. (22). In their report, the RCRP was thought to reflect the alert of peripheral defocus on retina. The RCRP was thought to be the mechanism by which orthokeratology controls myopia. However, to the best of our knowledge, no study has reported any relationship between retinal thickness or vasculature changes and corneal topography changes following orthokeratology. This study aimed to assess the correlation between the changes of retinal vascular density, retinal thickness, and corneal refractive power induced by wearing orthokeratology lens for 3 months.

## Materials and methods

### Subjects

A total of 30 children with myopia, aged 8–14 years, with corrected distance visual acuity (VA) better than 20/20, no cycloplegic subjective refraction and a spherical equivalent refractive error between −5.00 diopters (D) ~ −0.75 D in either eye, rule of astigmatism no greater than 1.50 D, anisometropia no greater than 1.50 D, and no previous experience of using contact lenses were included in this study. Prescreening ocular examinations, including anterior segment bio-microscopy, non-cycloplegic and subjective refraction, and retinal vasculature and thickness were conducted on all participants using OCTA (Optovue, Fremont, CA, United States). None of the participants had any other ophthalmic or systemic diseases. Only data from the right eye were analyzed. Age, sex, and spherical equivalent (SE) were recorded at baseline. The participants were instructed to return for study visits 1 day, 1 week, 1 month, and 3 months after wearing orthokeratology lenses. Ocular health, visual acuity, and corneal topography were re-assessed during these visits. The OCTA was operated only at baseline and 3 months after lens wearing, the other parameters of these two time points were used for statistical analysis accordingly.

Using the Lenstar LS900 (Haag-Streit, Bern, Switzerland), the axial length (AL) and biometric data, such as anterior chamber depth (ACD) and lens thickness (LT), were measured simultaneously at baseline and 3 months after wearing orthokeratology lens. Data analysis was performed on the first five readings with signal-to-noise ratios greater than 3 and intrasession differences of no greater than 0.02 mm.

Each participant and their parents were informed of all possible risks and written informed consent was obtained before commencing the study. The Institutional Human Research Ethics Committee of Artificial Eye Hospital, Wenzhou Medical University approved this study, and the study was conducted in accordance with the tenets of the Declaration of Helsinki. The study’s registration number on the Chinese Clinical Trial Registry web is ChiCTR1800019893.^1^

### Lens

In accordance with the manufacturer’s guidelines, all participants were fitted with orthokeratology lenses (Dreamlite Precorneal Ltd., Netherlands). The material used for lens fabrication was Boston XO (Dk ISO/Fat 141). The lenses were worn overnight. The total lens diameter (s), was approximately 10.5–10.9 mm with a 6 mm optic zone diameter. The lens was designed as 4-zoned. The clinical optometrist chose the spherical or tori lens based on the corneal parameters and the manufacturer’s fitting guidelines. Orthokeratology lens that fit well had a typical bullseye fluorescein pattern, with the lens movement between 1.0 mm and 1.5 mm. The OK lens was centered on the cornea. The corneal topography after treatment was not more than 1 mm decentration.

### Corneal topography

**Figure 1:**
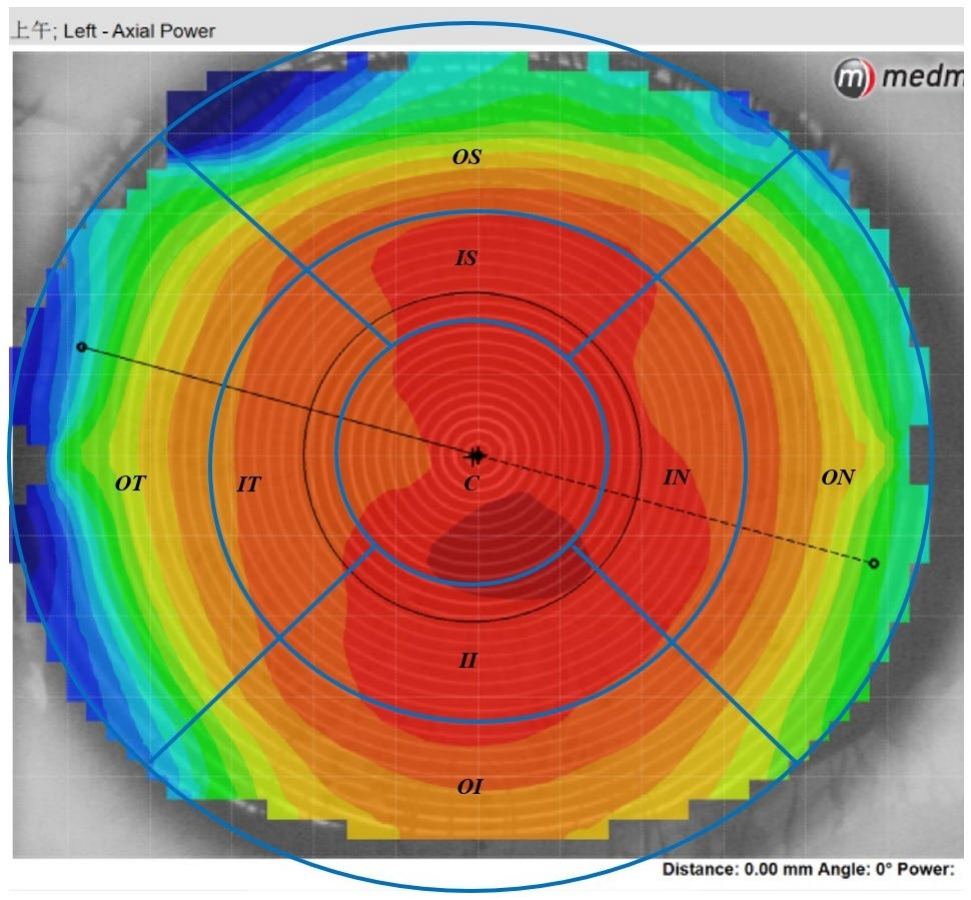
The axil corneal refractive power data diagram. The map was divided into 3 loops and 9 sectors by the blue lines in this schematic. The radius of center loop was up to 1.67 mm including 10 rings, the inner zone was from the 1.67–3.3 mm apart from the cornea apex including 11–20 rings, and the outer loop was from the 3.3–5.5 mm including 21–32 rings. Both of the inner and outer zone were divided into four sectors include superior, inferior, nasal and temple sectors. C, center; II, inner inferior; IT, inner temple; IN, inner nasal, IS: inner superior; OI, outer inferior; OT: outer temple; ON, outer nasal; OS, outer superior.

**Figure 2:**
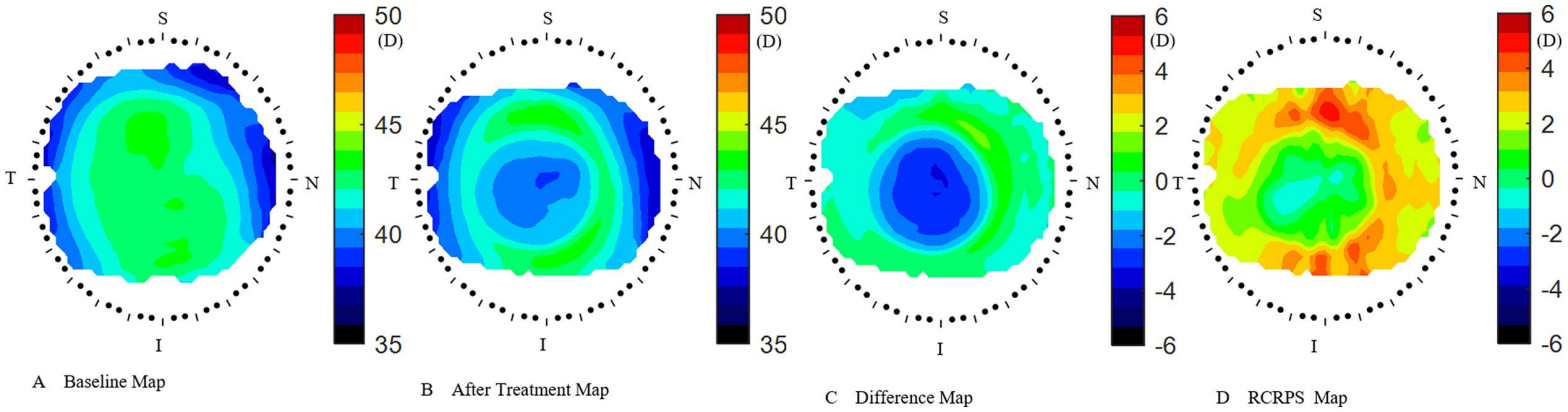
How to acquire the RCRPS. **(A)** the cornea axial map at baseline. **(B)** The cornea axial map at 3 months after treatment. **(C)** The difference map was acquired by subtracting the baseline map from the 3 months after-treatment map. **(D)** The RCRPS map was derived by subtracting the central cornea power from each point on the difference map. RCRPS, relative corneal refractive power shift. T: Temple, N: Nasal, S: Superior, I: Inferior. The corneal axial power scale was 35 ~ 50 diopter(D) in A and B, while −6 ~ 6D in C and D. RCRPS profile derived by taking the mean of each sector.

The Medmont E300 (Medmont International Pty. Ltd., Victoria, Australia) with the software of Medmont Studio 6 was used in this study. Each eye was imaged four times at each visit, and a score of >95% was recorded for statistical analysis. The axial corneal refractive power was automatically measured with chord lengths of approximately 11 mm. The corneal topography raw data within a radius of 5.5 mm including 32 rings were exported directly from the instrument, and then imported into the customized MATLAB software. The corneal axial keratometry raw data were used to reconstruct the cornea topography map, which was divided into 3 zones and 9 sectors. The radius of center zone was up to 1.67 mm including 10 rings, the inner zone was from the 1.67–3.3 mm apart from the apex including 11–20 rings, and the outer zone was from the 3.3–5.5 mm including 21–32 rings. Both of the inner and outer zones were divided into four sectors include superior, inferior, nasal and temple sectors. The sectors were named as center (C), inner inferior (II), inner superior (IS), inner temple (IT), inner nasal (IN), outer inferior (OI), outer superior (OS), outer temple (OT), outer nasal (ON). The detail of the corneal sectors is shown in the Figure 1. The detail of the data procession is found in previous literature (23, 24). In the research of Lee et al. (25), used the index of apex-periphery refractive power difference (ARPD) describe the corneal changes after wearing orthokeratology lenses. While Jiang et al., used the relative corneal refractive power shift (RCRPS) (22, 26, 27). In this study, we used RCRPS, which was defined as follows: compared with the baseline corneal topography, the posttreatment corneal topography, was used to acquire a difference map, and using the data of the difference map, the RCRPS was calculated by subtracting the central refractive power. The specific procedure of how to acquire the RCRPS is shown in the Figure 2.

### OCTA

Optical coherence tomography angiography scans were performed using an AngioVue XR Avanti device (Software V.2016.1.0.26 Optovue Inc., Fremont, CA, United States) in the angio-retina scan mode (6 × 6 mm) centered on the fovea. With a wavelength of 840 nm, the device can perform 70,000 A-scans per second using a super luminescent diode. By split-spectrum amplitude correlation angiography (SSADA), vascular information is optimized for the algorithm. The OCTA images define vascular density as the percentage of the image occupied by vascular tissue. Using the OCTA instrument, the superficial macular vascular density is automatically calculated. The detailed operation of OCTA had been described in many reports (28–31). Specifically, the OCTA images of 30 children were obtained preoperatively and 3 months after wearing orthokeratology lenses. Using this instrument, the superficial vascular image and retinal thickness were automatically obtained and exported for further analysis.

### Statistical analysis

Statistical analysis was performed using the statistical package for the social science (SPSS) software (version 20.0; IBM, Armonk, NY, United States). All variables underwent normality testing by Schapiro Wilk test. All results are expressed as the mean and standard deviation (SD). During preoperative and postoperative periods, the central refraction, corneal refractive power, and retinal density were compared using the paired t-tests for normally distributed data, and Mann–Whitney U tests were used for nonnormally distributed data. The relationships between the changes in corneal topography, retinal vasculature, and retinal thickness were analyzed by Spearman correlation analysis. A critical value of *p* of 0.05 was used to denote statistically significant results.

## Results

### Basic subjects’ characteristics

In total, 14 boys and 16 girls (mean age, 10.1 years; range 8–12 years) who underwent orthokeratology were enrolled in this study. All the children completed the 3-month follow-up. The spherical equivalent (SE) at baseline was −2.69 ± 0.96 D. Ocular parameters such as axial length (AL), anterior chamber depth (ACD), lens thickness (LT), corneal diameter of white to white (WTW), and anterior corneal Keratometry in the steepest and flattest meridians (KS, KF) were calculated using Lenstar (Table 1). After 3 months, compared with the results at baseline, ACD(3.21 ± 0.25 vs. 3.16 ± 0.27 mm), KS(43.82 ± 1.45 vs. 41.55 ± 1.28 mm), and KF (42.62 ± 1.45 vs. 40.37 ± 1.49 mm) decreased (*p* < 0.05), whereas the LT(3.38 ± 0.15 vs. 3.43 ± 0.18 mm) and WTW (12.05 ± 0.39 vs. 12.05 ± 0.38 mm) increased (*p* < 0.05). The AL showed a non-statistically significant increasing tendency (24.81 ± 0.86 vs. 24.84 ± 0.84 mm, *p* > 0.05). During the follow-up period, the corrected visual acuity gradually improved and stabilized at a naked eye visual acuity of 20/20 or higher after wearing lenses for 10 days to 1 month. Ocular health was checked at each follow-up visit, and there were no serious health events during the entire follow-up process.

**Table 1:** Biometric characteristics at baseline and 3 months after OK lens wearing.

Issue	Time			
	Baseline		M3	
	Mean	SD	Mean	SD
ACD (mm)	3.21	0.25	3.16	0.27
LT (mm)	3.38	0.15	3.43	0.18
AL (mm)	24.81	0.86	24.84	0.84
K1(D)	42.62	1.45	40.37	1.39
K2(D)	43.82	1.45	41.55	1.28
WTW (mm)	12.05	0.39	12.05	0.38
Pupil (mm)	5.15	1.02	4.62	0.85

AL, axial length; ACD, anterior chamber depth; LT, lens thickness; WTW, corneal diameter of white to white; KS, anterior corneal Keratometry in the steepest meridian; KF, anterior corneal Keratometry in the flattest meridians. All *p* value < 0.05.

### Retina microvascular density and retina thickness

The superficial retinal layer showed a central foveal macular vascular density (RVD)of 21.34 ± 6.39 at baseline and 20.21 ± 6.04 at 3 months after wearing an orthokeratology lens (*p* > 0.05). The total retinal layer thickness (RTC) showed central foveal macular thicknesses of 248.62 ± 13.73 at baseline and 249.17 ± 14.05 at 3 months after wearing orthokeratology lenses (*p* > 0.05). There was no significant change in microvascular density or retinal thickness after 3 months of wearing orthokeratology lens compared to that observed preoperatively (detailed data were presented in Tables 2, 3, all *p* values >0.05).

**Table 2:** Retina vascular density at baseline and 3 months.

Area	Base		3 M	
	Mean	SD	Mean	SD
C	21.34	6.39	20.21	6.04
II	49.93	4.25	49.00	4.92
IN	48.90	4.78	48.62	4.83
IS	50.83	4.42	49.76	5.28
IT	49.52	4.49	49.24	5.23
OI	49.41	3.34	48.29	3.42
ON	52.90	2.62	52.41	4.67
OS	49.55	2.81	49.07	2.64
OT	44.62	3.61	45.00	3.98

The location of retina: OS, superior retina; ON, outer nasal retina; OI, outer inferior retina; OT, outer temporal retina; IS, inner superior retina; IN, inner nasal retina; II, inner inferior retina; IT, inner temporal retina; C, central retina. All *p* value > 0.05.

**Table 3:** Retina thickness at baseline and 3 months.

Area	Base		3 M	
	Mean	SD	Mean	SD
C	248.62	13.73	249.17	14.05
II	317.21	10.58	317.31	11.08
IN	323.52	11.01	323.66	11.26
IS	324.83	10.95	325.17	10.66
IT	310.59	10.10	310.41	10.84
OI	271.48	10.35	272.66	10.62
ON	303.72	14.22	303.86	15.47
OS	288.83	11.21	289.28	10.53
OT	269.72	9.43	270.52	8.76

The location of retina: OS, superior retina; ON, outer nasal retina; OI, outer inferior retina; OT, outer temporal retina; IS, inner superior retina; IN, inner nasal retina; II, inner inferior retina; IT, inner temporal retina; C, central retina. All *p* value > 0.05.

### Corneal topography

**Figure 3:**
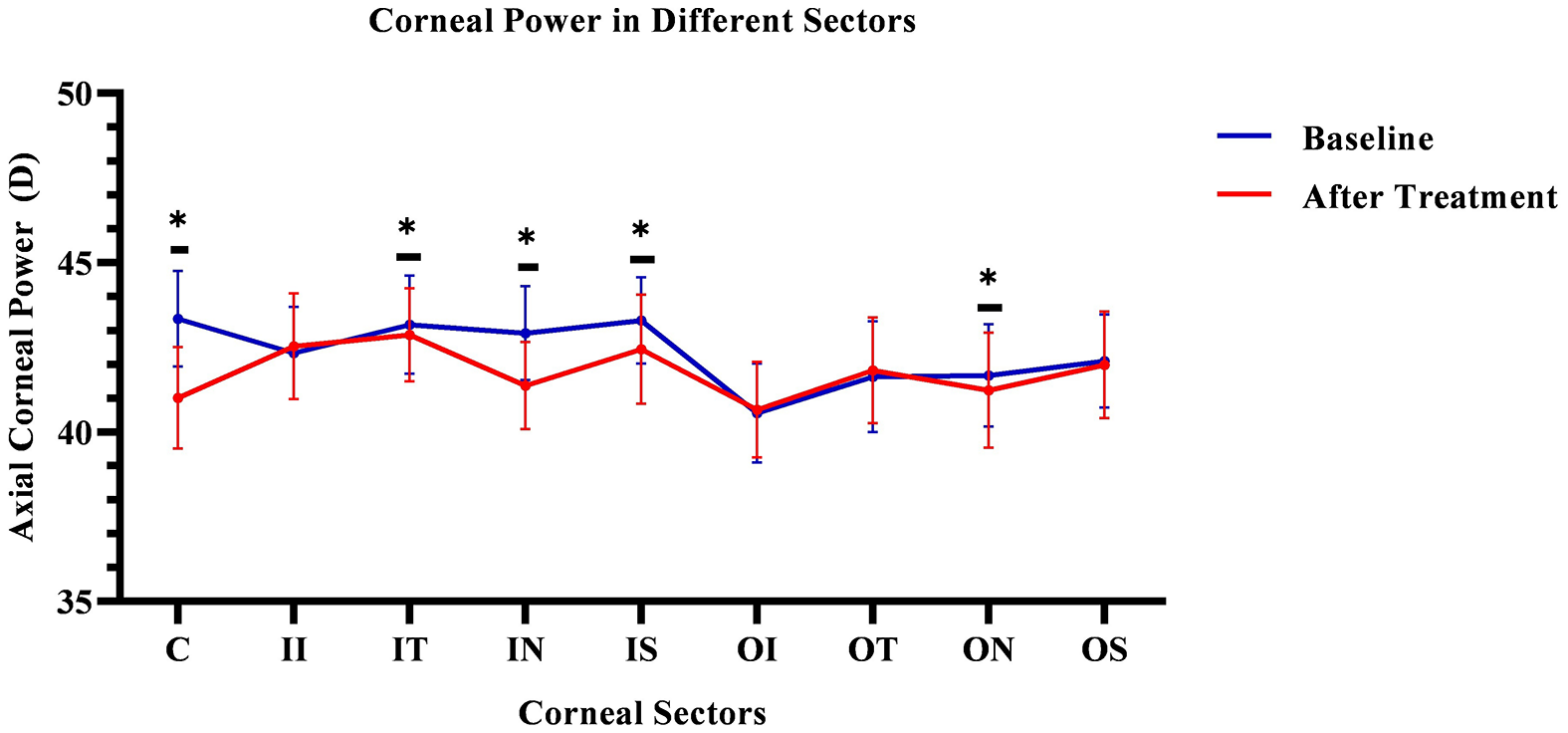
Comparing cornea Axial refractive powers in different sectors before and after OK lens wearing. The corneal refractive powers of the central zone (C) and peripheral central zones (IT, IN, IS, and ON) were significantly decreased after 3-month of wearing an orthokeratology lens (*p* < 0.05). The blue line represents the baseline data. The red line represents the data after treatment. The * mean the significant different between baseline and 3 months OK lens wearing. C, center; II, inner inferior; IT, inner temple; IN, inner nasal; IS, inner superior; OI, outer inferior; OT, outer temple; ON, outer nasal; OS, outer superior. The *means the significant different between the data of baseline and that of 3 months after OK lens wear.

**Figure 4:**
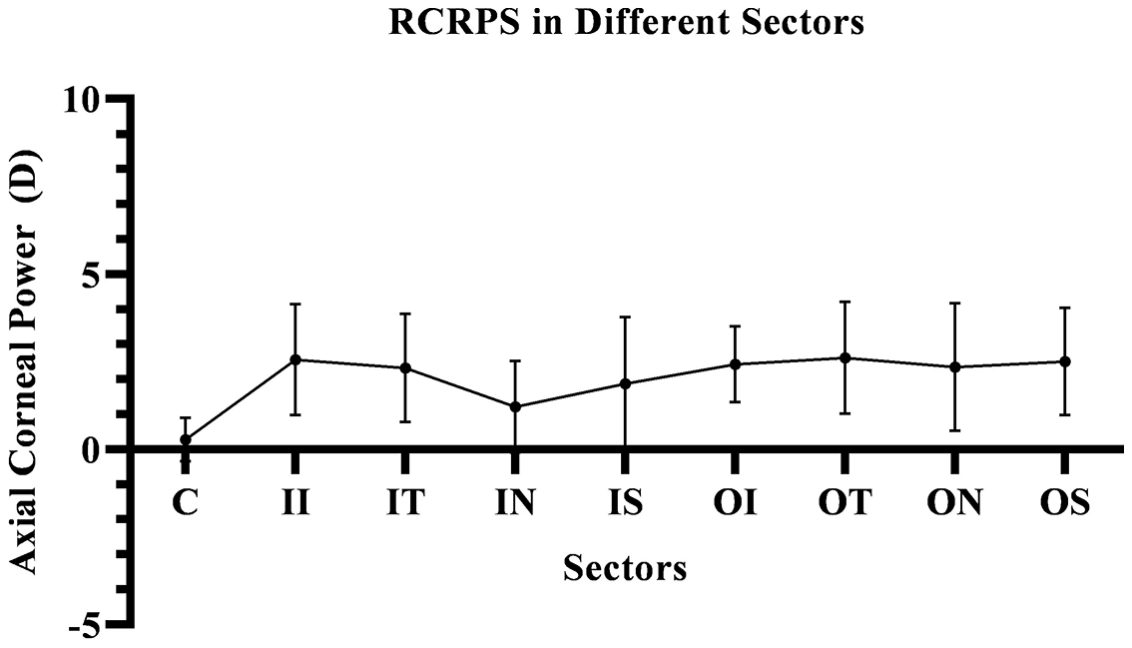
The RCRPS in Different Sectors. In the central cornea, the RCRPS was almost 0 and in other sectors, was positive data. C, center; II, inner inferior; IT, inner temple; IN, inner nasal; IS, inner superior; OI, outer inferior; OT, outer temple; ON, outer nasal; OS, outer superior; RCRPS, relative corneal refractive power shift.

The corneal axial refractive powers at different sectors in baseline were the steepest in the central cornea (43.35 ± 1.39D), and flattest in the outer inferior sector (40.56 ± 1.43D). After 3 months of lens wearing, the steepest data was 42.87 ± 1.35D in inner temple sector, and the flattest data was 40.66 ± 1.38D in the outer inferior sector. Comparing the cornea refractive power change, the corneal power significant decreased in the central cornea, and the inner zone including IN, IS, and IT after 3-month of lens wearing (all *p* < 0.05). While in the outer zone, the corneal power decreased only in the ON sector (*p* < 0.05). The remaining sectors had no significant change both in the inner and outer zones of the cornea (all *p* values >0.05). The comparing corneal data at baseline, and 3 months after lens wearing, are shown in Figure 3 and the RCRPS at every sector is shown in Figure 4. For RCRPS, the data of center cornea were almost clear focus, while the peripheral centra and peripheral cornea represents relative positive defocus. The detailed data of corneal axial refractive powers at baseline and 3 months, and the difference between the two visit points and the RCRPS are shown in the attachment 1.

### Correlation between RCRPS and retina change

**Figure 5:**
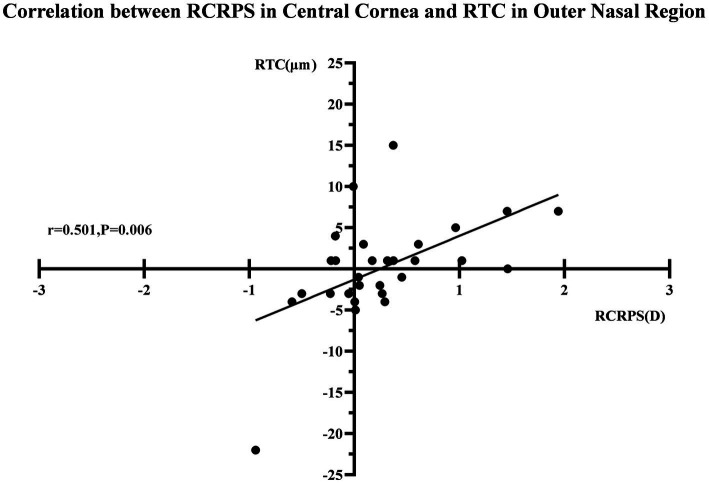
The correlation between RCRPS and RTC. The r was 0.501 and the *p* value was 0.006. RTC, retina thickness change; RCRPS, relative corneal refractive power shift.

**Figure 6:**
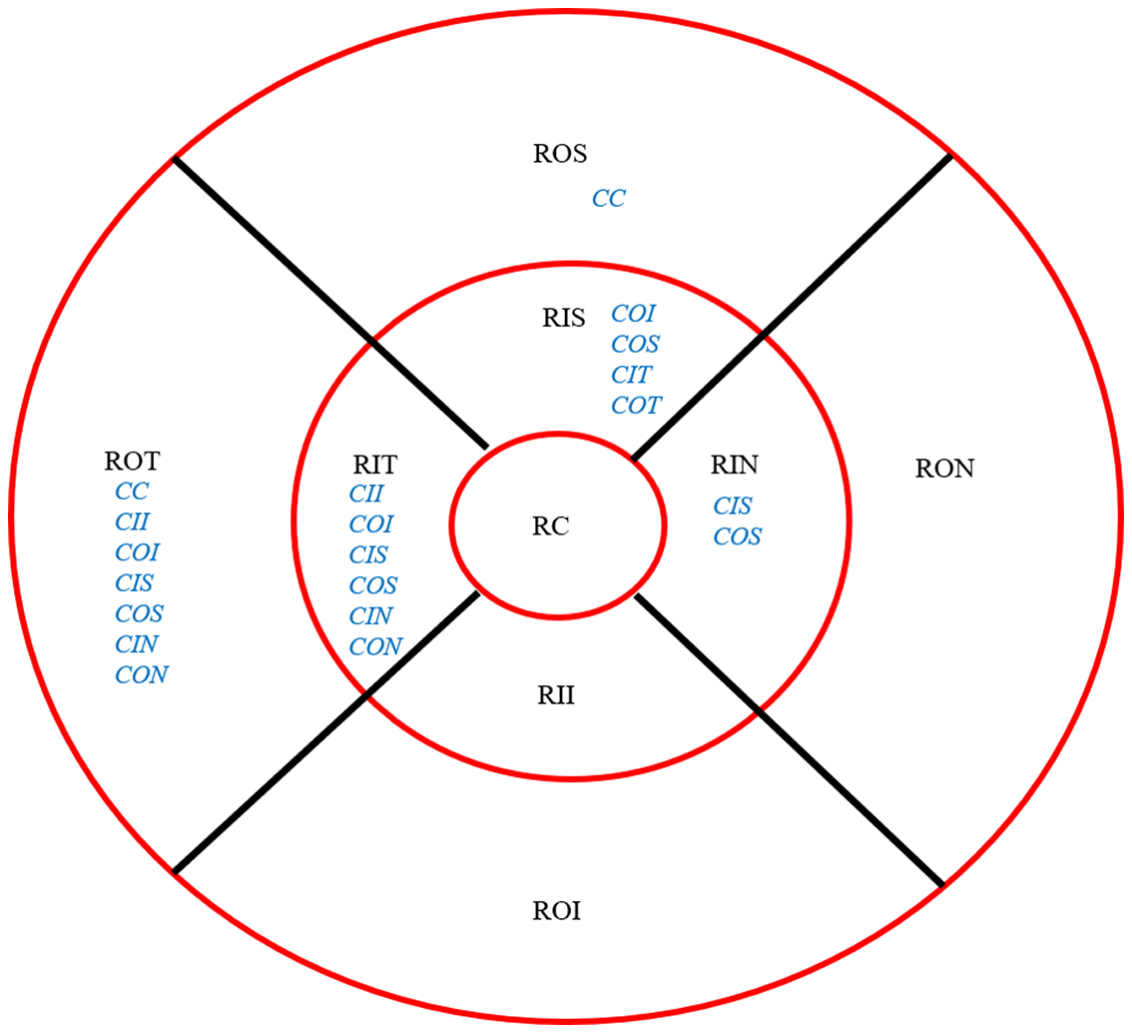
Overall Correlation between RCRPS and RVDC. The location of retina: ROS, superior retina; RON, outer nasal retina; ROI, outer inferior retina; ROT, outer temporal retina; RIS, inner superior retina; RIN, inner nasal retina; RII, inner inferior retina; RIT, inner temporal retina; RC, central retina. The RCRPS of cornea in different sectors: C, center; II, inner inferior; IT, inner temple; IN, inner nasal; IS, inner superior; OI, outer inferior; OT, outer temple; OS, outer superior. The blue words were for cornea and the black parts were for retina; RCRPS, relative corneal refractive power shift.

After 3 months, there was no significant correlation between the RCRPS and retina thickness changes at any zones except in the central cornea (C) and the outer nasal retina (ON) (*p* = 0.006, *r* = 0.501), shown in Figure 5. The results showed that the RCRPS in different sectors and changes in retinal vessel densities were correlated in multiple regions especially in the inner temple regions of the retina. Total correlations between the RCRPS and the regional RVDC are shown in Figure 6. Positive relationships were observed in many zones (*r* 0.375 ~ 0.548, all *p* value <0.05). At the temple retina, both the inner and outer zones, had positive correlations with more than five sectors of RCRPS. The correlation between RVDC in the temple retina and RCRPS was found to be significant for the nasal corneal region, but not for the temple cornea region, which was an interesting phenomenon.

Non-significant correlations were found in the inner and outer inferior regions, the outer nasal retina, and with any sectors of cornea RCRPS. The specific data are presented in the attachment 2.

## Discussion

In this study, regional correlations were observed between RCRPS (all the nine corneal sectors: C, IN, ON, IT, OT, IS, OS, II, and OI) and RVDC (retinal regions: OT, IT, IS, OS, and IN,). The relationships of the RCRPS and RVDC existed in the temple retina. The RCRPS in the central cornea had positive correlation with regional RTC only in the outer nasal region. Based on this phenomenon, RCRPS caused by orthokeratology had varied regional effect on the retina.

Orthokeratology is a safe and effective method of correcting low-to-moderate myopia by flattening the central cornea overnight with special design reverse geometry rigid gas permeable lenses (32, 33). In this study, as shown in Figure 3, the corneal power decreased significantly in center and the inner nasal, outer nasal, inner temple, and inner superior sectors of cornea after wearing orthokeratology lens. This is consistent with previous reports stating that the cornea is flatter in the center (34). While the cornea power did not significantly decrease in the inner inferior, inner inferior, outer superior, and outer temple regions. In the inner and outer zones, the inferior corneal power did not decrease significantly. We assume that because of the eyelid pressure during sleep, the inferior lens might lift and the cornea may not seal as effective as other regions. In the inner and outer zones, no corneal power increased in any sectors. This might be the average effect after segmenting cornea.

In this study, the RCRPS were all positive at all sectors, which mean that relative to the central area of the cornea, the diopter is larger, and its ability to converge light is stronger, that is, the relative myopic defocus state in cornea.

The RCRPS was reported by Wang et al. to describe the change of corneal power. In their reports, myopic control appears to be related to a larger RCRP modulation, specific 4.5D (22).The RCRPS related to the myopia progression in the report of Zhong et al., in which, the data were collected manually at 1 mm, 2 mm, 3 mm, and 4 mm away from the apex along the nasal, temporal, and inferior axes respectively, In the research by Jiang et al., based on the RCRPS, they described a new index of “Halfx” to quantify the RCRPS (27). All these researches, showed positive RCRPS, similar to our observations, although the instruments or the methods of RCRPS calculation were not exactly the same as those in our research.

Orthokeratology affects the cornea and the retina. Increasing research concerning fundus changes after orthokeratology exist; however, few studies have described retinal thickening or microvascular changes as a response to orthokeratology lens use. In this study, microvascular density decreased, whereas retinal thickness increased mildly; however, they were not statistically significant. The possible reasons for the non-significant changes in retinal thickness and vascular density before and after wearing OK lens in this study are speculated to be the relatively short follow-up period of only 3 months. A longer follow-up time may be required in future studies to investigate this further. Another possible reason is that the degree of myopia correction in our study was mild to moderate, which may result in weaker changes.

Recent studies have shown that the retina plays a crucial role in the development and progression of myopia. The retina is a complex and highly specialized neural tissue that contains several distinct layers of cells, including photoreceptors, bipolar cells, ganglion cells, and glial cells, which work together to convert light signals into electrical impulses that are transmitted to visual centers. The retina is also supplied with a rich vessel network that provide nutrients and oxygen. The fundus response is thought to be a part of the retina-choroid-sclera signaling pathway, which is one of the various theories proposed to explain myopia control interventions. Several neurochemicals and receptors have been implicated in the cascade of reactions within the retina, some of which are related to refractive error (35–37). New indices based on OCTA vessel density and retinal thickness were used in the adolescent myopia clinic work (38).

The retina operates independently and selectively regionally, which implies that different regions of the retina may have different levels of control over myopia. The regional difference of retinal morphology in the human eye has been described in previous studies. Several studies have shown that myopic atrophy is more likely to occur in the superior and temporal regions of the retina, which are also regions that exhibit significant regional variability in thickness and vascular density distribution.

These regional differences in the thickness and vascular density distribution of the retina may have important implications for the development of myopia. Specifically, in animal experiments, the choroid and retina of myopic animals have been found to become thinner during the myopia induction process (39, 40). Research of Read et al. found that the thickness of the choroid decreased significantly in myopic eyes compared to non-myopic eyes (41).

Peripheral retinal defocus is believed to play a key role in myopia control (42). Myopia defocus in the level of retina was thought stimulate the retina signal transitions and affect myopia progression. Based on animal studies, Mutti et al. found that peripheral hyperopic defocus is a risk factor for myopia onset in children and that peripheral myopic defocus inhibited myopia development (42–48). This may optimize the focus from the posterior globe. Several previous studies have shown that choroidal thickness increases after myopia control interventions (13, 48). Choroid thickening is believed to be primarily caused by peripheral myopic defocus due to corneal topography changes after wearing orthokeratology lens (15, 49, 50).

A study by Wallman et al. suggested that local defocus may control the local eye growth and progression of myopia, based on the assumption that the peripheral retina defocuses asymmetrically (40). A study conducted by Wallman et al. found that the peripheral retina plays a critical role in regulating eye growth and preventing myopia (51). They found that the peripheral retina sends signals to the brain that inhibit the growth of the eye, which helps to maintain the proper balance between the length of the eye and its optical power. Another study by Smith et al. found that the central retina may also play a role in myopia development, as it is responsible for fine-tuning the focus of the eye (52).

Despite the lack of statistical difference in the absolute data of RVD and RT, there was a positive and mild to medium correlation between RCRPS and RVDC in this study. As we know, orthokeratology lenses can cause significant changes in corneal topography and refractive power zones, which can lead to retinal myopia defocus. Retinal myopia defocus was thought to be the mechanisms by which orthokeratology controls myopia. In this study, the changes of retinal vascular density in many regions had significant correlation with the relative defocus of corneal change. Anatomical differences between the nasal and temporal sides of the retina may account for this change.

The correlation in retinal vascular density in the temporal and superior regions and the multi-position RCRPS suggest that the changes in the retina in response to corneal changes vary regionally. Myopia arc, posterior staphyloma, and choroidal atrophy are prone to occur in the temporal region. Therefore, such correlations may imply that under the induction of optical signals, the vascular density on the temporal side of the retina might change correspondingly, which may delay the occurrence of choroidal atrophy and reduce or delay the occurrence of myopia arc.

In our study, there was a significant association between the outer superior and temple retinal vascular and nasal topographic changes. This localized association may be due to the photoelectric or photochemical reaction of the retina caused by the direction of light. The electrophysiological responses at the retinal center and periphery may alter myopia progression (53). Active changes occur in the retina under different light refraction due to corneal morphology changes after orthokeratology treatment. More light was refracted on the temple side because there was more open space.

According to this study, the correlation was in the inner temple and outer temple retina superior retina, as myopia atrophy often occurs in the temple retina and inferior retina around the optic disc. Myopia may have an atrophic arc on the temporal side of the optic disc, which corresponds to the nasal side of the macula anatomically. Therefore, in this study, the refraction of light from the cornea showed no such correspondence, or the correspondence exists but the response of retina vascular density was focus on the temple regions. If the position and amount of the RCRPS correspond to the retinal changes in the corresponding region, the position and amount must be confirmed, which needs further research.

### Limitations

This study has several limitations. First, follow-up period was short. A longer follow-up period should be used in future studies. Second, the sample size was relatively small. Future studies with lager sample sizes are warranted.

## Conclusion

As an explorative study to assess RCRPS in relation to RVDC and RTC, the results showed that RCRPS correlated with the RVDC mostly in the inner temple region after a short time of OK lens wearing. OK lens causes changes in the cornea regionally, which directly leads to regional changes in the corneal topographic map. The changes of the cornea lead to corresponding defocusing at the retinal level, which may also lead to regional changes in the fundus. The regional changes in retinal vascular density may be related to retinal signal transduction. Such a chain reaction may be a mechanism for myopia control.

## Data availability statement

The original contributions presented in the study are included in the article/Supplementary material, further inquiries can be directed to the corresponding author.

## Ethics statement

The studies involving human participants were reviewed and approved by Institutional Human Research Ethics Committee of Artificial Eye Hospital, WenZhou Medical University. Written informed consent to participate in this study was provided by the participants' legal guardian/next of kin.

## Author contributions

QL, RC, AX, LM, and WZ collected and analyzed the data. YL, WL, and WJ designed and supervised the study. YL analyzed the data and wrote the manuscript. All authors contributed to the article and approved the submitted version.

## Funding

This study was supported by the Science and Technology Project of Wenzhou (nos. Y20190630, Y2020348).

## Conflict of interest

The authors declare that the research was conducted in the absence of any commercial or financial relationships that could be construed as a potential conflict of interest.

## Publisher’s note

All claims expressed in this article are solely those of the authors and do not necessarily represent those of their affiliated organizations, or those of the publisher, the editors and the reviewers. Any product that may be evaluated in this article, or claim that may be made by its manufacturer, is not guaranteed or endorsed by the publisher.

## Footnotes

^1^ http://www.chictr.org.cn/index.aspx
