# Supplementary material for: The correlation between modifications to corneal topography and changes in retinal vascular density and retinal thickness in myopic children after undergoing orthokeratology
Source: Front Med (Lausanne). 2023 Jun 29;10:1166429. doi: 10.3389/fmed.2023.1166429 (PMC10338965; doi:10.3389/fmed.2023.1166429)
Supplement: Supplementary file 1 [file Table_1.DOCX]

The detailed data of corneal axial refractive powers (baseline and 3 months， the difference of the two time and the RCRPS)

| Location | Base mean | Base SD | M3 mean | M3 SD | Diff mean | Diff SD | RCRPS mean | RCRPS SD |
| --- | --- | --- | --- | --- | --- | --- | --- | --- |
| CC | 43.34721 | 1.392013 | 41.01287 | 1.477008 | -2.33434 | 0.955886 | 0.281214 | 0.612057 |
| CII | 43.28877 | 1.253292 | 42.43952 | 1.58487 | -0.84925 | 0.943469 | 1.868156 | 1.873703 |
| CIT | 43.16882 | 1.424952 | 42.86533 | 1.354875 | -0.3035 | 0.754045 | 2.323829 | 1.517101 |
| CIN | 42.92041 | 1.365295 | 41.36718 | 1.266436 | -1.55323 | 0.582217 | 1.214909 | 1.283982 |
| CIS | 42.32735 | 1.342322 | 42.53357 | 1.53579 | 0.206212 | 0.675821 | 2.561357 | 1.560923 |
| COI | 42.09822 | 1.350624 | 41.98102 | 1.544437 | -0.1172 | 0.444694 | 2.503105 | 1.511833 |
| COT | 41.66896 | 1.483996 | 41.23242 | 1.673231 | -0.43654 | 0.744658 | 2.35105 | 1.784757 |
| CON | 41.63167 | 1.605496 | 41.82007 | 1.536344 | 0.188399 | 0.737554 | 2.612619 | 1.563681 |
| COS | 40.55964 | 1.431663 | 40.6569 | 1.384645 | 0.097254 | 0.452287 | 2.433717 | 1.074976 |
