# Supplementary material for: The correlation between modifications to corneal topography and changes in retinal vascular density and retinal thickness in myopic children after undergoing orthokeratology
Source: Front Med (Lausanne). 2023 Jun 29;10:1166429. doi: 10.3389/fmed.2023.1166429 (PMC10338965; doi:10.3389/fmed.2023.1166429)
Supplement: Supplementary file 2 [file Table_2.DOCX]

| corneal location | retina location | r | P |
| --- | --- | --- | --- |
| CC | ROS | 0.390416 | 0.03627 |
| CC | ROT | 0.380203 | 0.0419 |
| CII | RIT | 0.429757 | 0.01998 |
| CII | ROT | 0.411144 | 0.026711 |
| CIT | RIS | 0.423341 | 0.022121 |
| CIN | RIT | 0.374647 | 0.045243 |
| CIN | ROT | 0.37748 | 0.043512 |
| CIS | RIT | 0.547884 | 0.002094 |
| CIS | RIN | 0.406587 | 0.028613 |
| CIS | ROT | 0.525749 | 0.0034 |
| COI | RIS | 0.399808 | 0.031645 |
| COI | RIT | 0.451998 | 0.013832 |
| COI | ROT | 0.56065 | 0.001559 |
| CON | RIS | 0.417891 | 0.024085 |
| CON | RIT | 0.430745 | 0.019665 |
| CON | ROT | 0.518818 | 0.003931 |
| COS | RIS | 0.485765 | 0.007553 |
| COS | RIT | 0.401584 | 0.030827 |
| COS | RIN | 0.389264 | 0.036872 |
| COS | ROT | 0.453471 | 0.013488 |

The total correlations between the RCRPS and the regional RVDC
